# Effectiveness of cholinium amino acid ionic liquids for the extraction of phytonutrients from palm oil

**DOI:** 10.1098/rsos.241719

**Published:** 2025-04-09

**Authors:** S. Y. Chong, K. Y. Khaw, R. F. S. Lee

**Affiliations:** ^1^ School of Pharmacy, Monash University Malaysia, Bandar Sunway, Selangor 47500, Malaysia

**Keywords:** ionic liquids, palm oil, tocotrienol, tocopherol, vitamin E, functional food

## Abstract

Palm oil is the most widely consumed vegetable oil globally, owing to its high yield and cost-effectiveness. Beyond its primary use, palm oil is rich in valuable phytonutrients such as tocols (tocopherols and tocotrienols) and carotenoids, which hold significant economic value. Solvent extraction is a promising method to recover phytonutrients from palm oil; however, the use of flammable and toxic organic solvents raises concerns about environmental sustainability and infrastructure costs, necessitating the development of safer and more efficient solvent systems. This study investigates the potential of ionic liquids (ILs), specifically cholinium (CH) amino acid (AA) ILs ([CH][AA] ILs), as environmentally friendly and biocompatible solvents for extracting tocols and β-carotene from crude palm oil. Six [CH][AA] ILs, derived from alanine, serine, proline, glycine, leucine and lysine, were synthesized and evaluated for their extraction efficiency. Most of these ILs demonstrated significant efficacy in extracting tocotrienols and tocopherols, with the lysine-based IL showing the highest performance, extracting over 200 mg kg⁻¹. In terms of extraction efficiency, the ILs ranked as follows: lysine > proline > glycine > serine > leucine > methanol (not an IL) > alanine. Notably, none of the [CH][AA] ILs were able to extract β-carotene, highlighting their selectivity for tocols.

## 1. Introduction

Palm oil is extracted from the fresh mesocarp of the oil palm fruit (*Elaeis guineensis*) [1]. Approximately 90% of global palm oil production serves the food industry, where it is extensively used as cooking oil, in margarine and shortenings, and for manufacturing nutritional supplements [2]. Malaysia and Indonesia dominate palm oil production, collectively accounting for over 85% of the global supply [3]. With global demand for palm oil projected to reach 240 million tons by 2050, its applications have expanded beyond food to non-food sectors like biodiesel production, further emphasizing its versatility [4].

Although phytonutrients represent a minor component of palm oil, they play a crucial role in maintaining its stability and oxidative resistance. These phytonutrients include carotenoids, tocopherols, tocotrienols (commonly referred to as tocols), sterols, squalene, phospholipids, coenzyme Q10 and polyphenols [5]. Estimated quantities of some key phytonutrients are presented in table 1. Recently, there has been growing research interest in these compounds owing to their numerous health benefits, including antioxidative, anti-cancer, anti-inflammatory and neuroprotective properties [5]. Economically, the global phytonutrient market was valued at $4.7 billion in 2024 and is expected to grow significantly, reaching $6.64 billion by 2028 [7]. This rising demand underscores the need for efficient methods to extract and purify phytonutrients for commercial applications.

**Table 1.** Estimated content of common phytonutrients found in palm oil (adapted from [6]).

phytonutrients	values (mg kg^−1^)
phytosterols	270–800
squalene	200–540
carotenoids	500–700
tocols	600–1000

For the extraction of phytonutrients from palm oil, various methods have been investigated, including molecular distillation, short-path distillation, adsorption (column chromatography and flash chromatography), membrane processing, solvent extraction and supercritical fluid extraction [5]. Molecular and short-path distillation provide selective extraction capabilities but require substantial capital investment and operate at extreme temperatures of up to 250°C, which may degrade phytonutrients [2]. While membrane processing is energy- and cost-efficient, its application is limited by the high viscosity of crude palm oil (CPO), necessitating elevated feed temperatures and operating pressures to improve permeate flux. Adsorption or chromatographic separation, which can be conducted at room temperature and atmospheric pressure, is well suited for heat-sensitive compounds but is relatively time-intensive owing to the viscous nature of CPO [5]. Flash chromatography, a high-throughput adaptation of traditional column chromatography, improves separation efficiency using air pressure but requires significant initial capital investment [8].

Another approach to phytonutrient extraction from edible oils is solvent extraction. This method involves extracting compounds from palm oil using solvents that are immiscible with oil and have a high affinity for the target compounds. Various solvents such as alcohols, ethyl acetate and supercritical carbon dioxide have been used for this purpose [9]. However, these solvents have limitations, including high toxicity, flammability or requiring significant capital investment for equipment set-up. Ionic liquids (ILs) are a potential alternative solvent for solvent extraction. These are molten salts that remain in a liquid state at temperatures below 100°C, consisting of combinations of large, asymmetrical organic cations paired with either organic or inorganic anions [10]. These salts have beneficial physicochemical properties like excellent chemical and thermal stability, non-flammability, low volatility and the ability to be tuned to be task-specific based on the large number of ion combinations that can form ILs [10].

**Figure 1.**
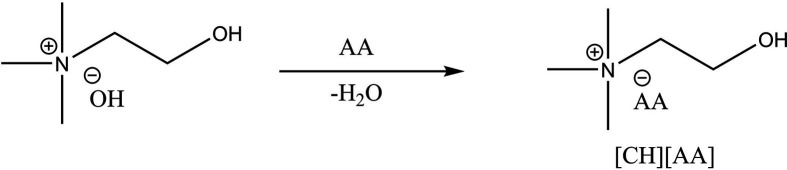
Synthesis of cholinium amino acid ionic liquids ([CH][AA] ILs).

However, the use of ILs for food-related applications requires careful selection of ions to ensure they are safe for human consumption [11–13]. For phytonutrient extraction from palm oil, Abdul Hadi *et al*. [2] investigated cholinium carboxylic acid-based ILs, which are biocompatible and relatively effective for extracting tocols. However, the hydrophilic nature of short-chain carboxylic acid anions may limit their efficiency in extracting hydrophobic phytonutrients like tocols and carotenes. By contrast, cholinium amino acid-based ILs ([CH][AA] ILs) offer a promising alternative. These ILs combine the biocompatibility of choline—a non-toxic micronutrient serving as the cation—with amino acids, the building blocks of proteins, as anions. The diverse physicochemical properties of amino acid residues may further enhance extraction selectivity [14]. Another issue in existing studies is the high viscosity of ILs and the relatively high melting point of CPO, which often necessitates the use of organic solvents to liquefy the oil and reduce viscosity. However, the toxicity and flammability of these additional solvents present significant concerns. Here, we aim to address these gaps by synthesizing a series of [CH][AA] ILs and examining their selectivity and extraction efficiency for tocols and carotenes from CPO, without the need for additional solvents (figure 1).

## 2. Experimental

### 2.1. Material and methods

Choline hydroxide solution (46 w/w% in H_2_O), l-alanine (≥ 98%), l-proline (≥ 99%), l-serine (≥ 99%), l-leucine (≥ 99%), l-lysine (≥ 99%) and l-glycine (≥ 99%) were purchased from Sigma Aldrich (USA). Tocotrienol secondary standards and CPO were kindly provided by SOP Green Energy Sdn. Bhd. (Sarawak, Malaysia). Acetonitrile, hexane and methanol (≥ 95%) were purchased from Macron (Norway).

### 2.2. Synthesis of cholinium amino acid ionic liquids

Ionic liquids ([CH][AA] ILs) were synthesized using a modified procedure based on Sadanandan *et al*. [14]. A total of 32 mmol of amino acid was dissolved in Milli-Q water (volume adjusted based on the solubility of the amino acid) with magnetic stirring for 1 h at room temperature. The solution was neutralized by adding 30 mmol of choline hydroxide dropwise, followed by stirring for at least 12 h at room temperature. Water was removed using a rotary evaporator. A mixture of acetonitrile and methanol (9 : 1 v/v) was added to the crude product, stirred for 1 h and filtered. The filtrate was evaporated using a rotary evaporator, and the resulting light brown viscous liquid was dried in a vacuum oven at 50°C for 2 days. Further drying was performed using high vacuum with a rotary vane pump over a Schlenk line. The ILs were stored in glass bottles and kept in the dark for subsequent experiments. ILs were characterized using ^1^H nuclear magnetic resonance (NMR) spectroscopy (Bruker Avance Neo 300 MHz, deuterated methanol as the solvent) and Fourier transform-infrared (FTIR) spectroscopy (Agilent Cary 360). NMR chemical shift values and FTIR wavenumbers are provided in electronic supplementary material, characterization.

### 2.3. Extraction procedure

Five grams of CPO were mixed with an equal weight of solvent (1 : 1 w/w ratio) and placed in a shaker incubator at 200 rpm and 60°C for 1 h to liquefy the CPO and reduce the viscosity of the solvent–CPO mixture. The mixture was then centrifuged at 2000 rpm for 1 min and allowed to stand for 1 h at 60°C to form two layers. The CPO formed the top layer, while the IL/methanol constituted the bottom layer (reversed when methanol was used as the solvent). Both layers were separated and stored at 4°C for β-carotene and tocol analysis.

### 2.4. Recycling procedure

The post-extraction IL layer was mixed with ultrapure water at a 1 : 1 ratio. The mixture was centrifuged at 2000 rpm for 1 min and allowed to stand for 1 h to form two layers. The top layer, containing water-immiscible substances such as tocols, was removed. The bottom layer, consisting of the IL–water mixture, was recovered. Water was removed via rotary evaporation to recycle the IL, which was then used for a second round of extraction. Tocol content in the recycled IL was analysed before and after the second extraction cycle.

### 2.5. Tocols content analysis

Tocol content was analysed using high performance liquid chromatography (HPLC), following a slightly modified method from Yap *et al*. [15]. An Agilent 1200 HPLC system equipped with a fluorescence detector was employed, with excitation and emission wavelengths set at 296 and 330 nm, respectively. Separation was achieved using a Phenomenex Luna C18 column (5 µm, 250 mm × 4.6 mm internal diameter) paired with a guard column. The analysis was conducted with a 20 µl injection volume, a 30 min isocratic run and a mobile phase consisting of 99.95% methanol and 0.05% water, maintained at a flow rate of 1 ml min^−1^. Quantification was performed using a calibration curve prepared from tocotrienol-rich fraction secondary standards at concentrations of 1.56, 3.13, 6.25, 12.5, 25, 50 and 100 mg kg^−1^. These standards contained 50.3% palm tocotrienols and tocopherols, with the composition comprising 4.4% δ-tocotrienol, 18.6% γ-tocotrienol, 12.2% α-tocotrienol, 1.5% β-tocotrienol and 13.5% α-tocopherol. Standard stock solutions were stored in amber glass vials and used within three months. Sample preparation involved thorough mixing of the IL solvent layer, followed by a two-step dilution: an initial 3× dilution in dimethyl sulfoxide and a subsequent 33× dilution in methanol, resulting in a total dilution factor of 99×. The diluted sample was centrifuged at 1500 rpm for 1 min to remove any undissolved particles. A 500 µl aliquot of the supernatant was then transferred into HPLC vials for analysis.

Peak resolution was calculated using the formula:

.Rs = 2 (RT2 − RT1) / (W1 + W2)

— Rs = resolution
— RT1, RT2 = retention times of the two peaks (with RT2 > RT1)
— W1, W2 = baseline peak widths

### 2.6. β-carotene content determination

The β-carotene content in CPO was quantified using ultraviolet-visible spectroscopy (SpectraMax ABS Plus, Molecular Devices) following a scaled-down Malaysian Palm Oil Board (MPOB) test method [16]. Approximately 0.02 g of CPO, weighed to the nearest 0.001 g, was dissolved in 5 ml of hexane. Absorbance was measured at 446 nm, and samples were diluted to achieve readings between 0.2 and 0.8 optical density (OD). The total β-carotene content was calculated using the formula:


$$x=\frac{383\;E}{IC},$$

where *x* is the total β-carotene content in oil sample (mg kg^−1^), *E* is the difference in absorbance between oil sample and hexane (OD), *I* is the path length of the cell (1 cm) and *C* is the concentration of oil sample (g 100 ml^−1^).

### 2.7. Statistical analysis

All extractions and analyses were performed in triplicate, with results expressed as mean ± s.d.

## 3. Results and discussion

### 3.1. Tocol content by ionic liquid extraction from crude palm oil

**Figure 2.**
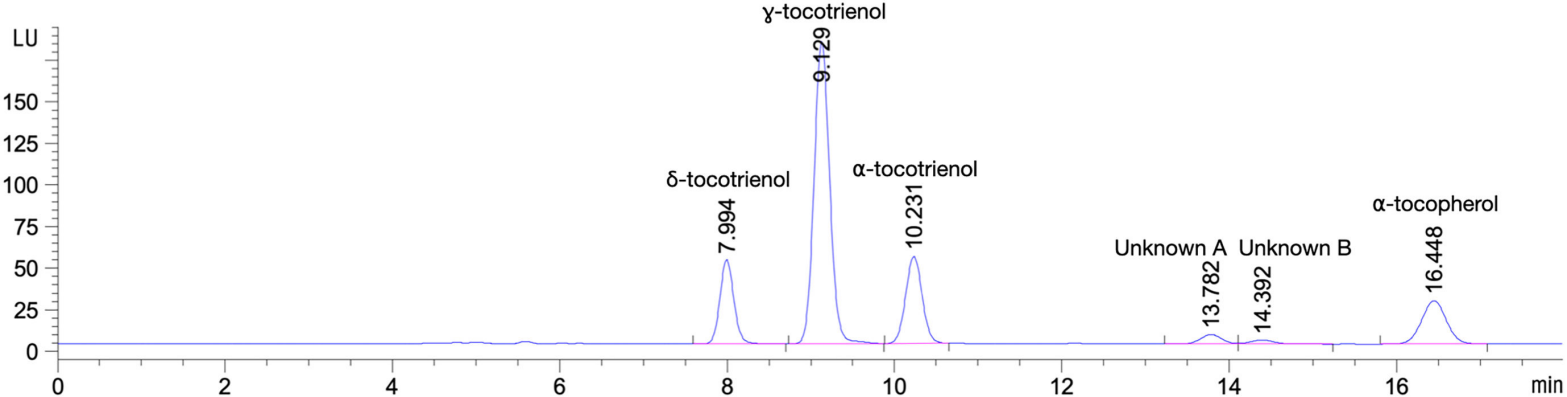
HPLC chromatogram of 100 mg kg^−1^ secondary tocotrienol standards using fluorescence at Ex = 296 and Em = 330.

To calculate tocotrienol concentrations from ILs, we adopted the method described by Yap *et al*. [15], using a tocotrienol-rich fraction as a secondary standard. Well-resolved peaks were obtained for δ-tocotrienol, γ-tocotrienol, α-tocotrienol and α-tocopherol (figure 2). The separation resolutions were 5.93 (δ-tocotrienol and γ-tocotrienol), 5.29 (γ-tocotrienol and α-tocotrienol), 14.53 (α-tocotrienol and unknown A) and 6.55 (unknown B and α-tocopherol). Within a range of 1.56–100 mg kg^−1^ of tocotrienol secondary standards, we obtained a linearity of *R*^2^ = 0.09961–0.9998 (electronic supplementary material, figure S1). The limit of detection (LOD) for δ-, γ- and α-tocotrienol are 0.581, 0.852 and 0.341 mg kg^−1^, respectively, whereas for limit of quantitation (LOQ) were 1.76, 2.58 and 1.03 mg kg^−1^, respectively. For α-tocopherol, we obtained an LOD of 0.272 mg kg^−1^ and an LOQ of 0.823 mg kg^−1^. Overall, the separation resolution and sensitivity of our system are higher than those reported by Yap *et al*. [15], probably owing to advancements in sensitivity and resolution of HPLC in the past 20 years.

Most studies on IL extraction of phytonutrients from CPO employ solvents like hexane and methanol to reduce the viscosity of the oil and ILs, respectively [2,8,17]. By contrast, this study used neat extractions with CPO and ILs, avoiding the use of additional organic solvents, which are often flammable and toxic. However, performing neat extractions posed significant challenges as CPO is solid at room temperature, and the ILs exhibit high viscosity owing to their extensive hydrogen bonding network [18]. To address these issues, all extractions were conducted at 60°C to ensure that CPO remained in a liquid state and to reduce the viscosity of the ILs. After extraction, some ILs partially solidified upon cooling. To ensure representative sampling for tocol measurements, the IL samples were thoroughly homogenized. Dilutions were then performed, first in dimethylsulfoxide and subsequently in methanol, to ensure that all phytonutrients were uniformly mixed before HPLC analysis.

The concentrations (mg kg⁻¹) of individual tocol components extracted by the ILs are summarized in table 2. Overall, the extraction efficiency of tocols follows the order: [CH][lysine] > [CH][proline] > [CH][glycine] > [CH][serine] > [CH][leucine] > methanol > [CH][alanine].

**Table 2.** Extraction of individual tocols (mg kg^−1^) from CPO by synthesized ILs.

[CH][AA]IL	individual tocols concentration (mg kg^−1^)				total values of all tocol subtypes (mg kg^−1^)
	δ-tocotrienol	γ-tocotrienol	α-tocotrienol	α-tocopherol	
**methanol**	9.935 ± 3.461	18.784 ± 8.850	15.025 ± 2.744	17.645 ± 2.432	61.389
**[CH][alanine]IL**	6.164 ± 1.152	13.200 ± 4.533	10.151 ± 1.939	15.938 ± 4.307	45.453
**[CH][serine]IL**	25.022 ± 0.808	74.486 ± 2.755	23.914 ± 0.516	34.057 ± 0.164	157.479
**[CH][proline]IL**	30.568 ± 0.829	90.756 ± 1.823	31.656 ± 0.894	37.852 ± 0.854	190.832
**[CH][leucine]IL**	26.821 ± 2.349	58.056 ± 6.572	19.727 ± 3.381	18.973 ± 3.614	123.577
**[CH][lysine]IL**	34.864 ± 2.255	109.457 ± 8.770	40.201 ± 3.293	53.220 ± 8.684	237.742
**[CH][glycine]IL**	26.297 ± 0.377	79.135 ± 0.391	30.050 ± 0.604	43.828 ± 0.753	179.310

The overall hydrophobicity/hydrophilicity of solvents plays a role in the extraction of natural products. For [CH][AA] ILs, the hydrophobicity/hydrophilicity differences are contributed by the amino acid anion. Hydrophobicity/hydrophilicity of amino acids can be determined by hydropathy scales such as the Kyte–Doolittle scale [19], where the higher the number, the more hydrophobic the amino acid is. We observed that, in general, the extraction efficiency of tocols with [CH][AA] ILs was proportional to the hydrophilicity of amino acid anion. The amino acids used in this study in descending order of hydrophobicity and their hydropathy index are leucine (3.8), alanine (1.8), glycine (−0.4), serine (−0.8), proline (−1.6) and lysine (−3.9). As shown in table 2, the highest extraction efficiency was seen in [CH][AA] ILs with hydrophilic amino acid lysine, and the lowest extraction was seen in hydrophobic side-chain amino acids alanine and leucine.

In addition, molecular interactions such as hydrogen bonding and van der Waals interactions can also be involved in determining interactions between solvents. Tocols contain either a saturated phytyl side chain in tocopherols or unsaturated isoprenoid side chain in tocotrienols that can take part in van der Waals interactions with solvents. These interactions increase with increasing carbon chain lengths in molecules. When comparing extraction efficiency between hydrophobic amino acid [CH][AA] ILs, we found that [CH][leucine] IL extracted 123.58 mg kg^−1^ of tocotrienol and [CH][alanine] IL extracted 45.45 mg kg^−1^. This can be explained by the difference in carbon chain length with leucine having a branched C4 side chain and alanine having a C1 side chain [20].

In tocols, the hydroxyl group and oxygen group of the chromanol ring can serve as hydrogen bond donors and acceptors, respectively. For the [CH][AA] ILs, the choline group contains both hydrogen bond donors and acceptors, and the anion pair, which consists of different amino acid types, has different extents of hydrogen bonding capacity based on the different R groups present. Among the amino acids used for making ILs, both lysine and serine can form hydrogen bonds. Overall, the high extraction efficiency of [CH][lysine] IL is probably owing to the combination of hydrogen bonding, van der Waals interaction from the C4 chain and its higher polarity [20].

**Figure 3.**
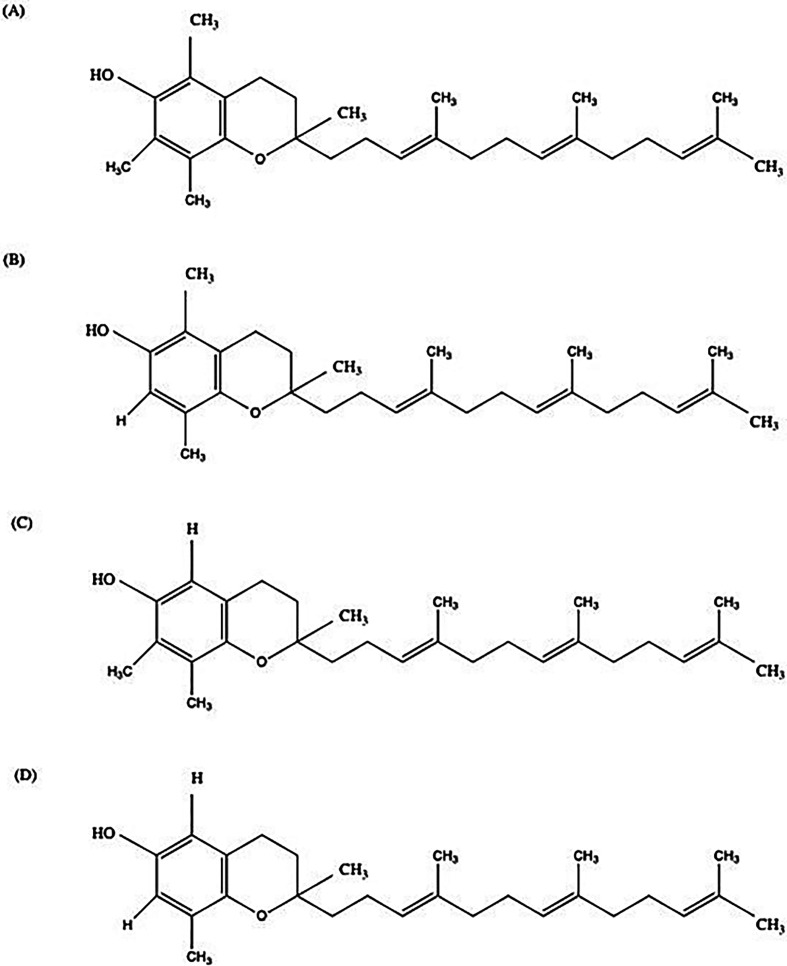
Structures of the four types of tocotrienols: (A) α-tocotrienol, (B) β-tocotrienol, (C) γ-tocotrienol and (D) δ-tocotrienol.

Regarding the extraction by tocol subtype, the ILs were more effective at extracting δ-tocotrienol compared with other tocol subtypes observed as detailed in table 1. This may stem from the variations in hydrophobicity among the tocotrienol subtypes, with δ-tocotrienol being the least hydrophobic owing to its single methyl group substitution on the dihydrocoumarin head, compared with two in γ-tocotrienol and three in α-tocotrienol. The structures of the various tocotrienol types are depicted in figure 3. Consistent with our findings that ILs with lower hydrophobicity were more effective in tocol extraction, the less hydrophobic tocotrienol subtypes appeared to be extracted more efficiently using these ILs. Among the tocotrienol subtypes, δ-tocotrienol has been shown to have particular potency in cancers, where it was shown to have higher angiogenesis inhibitory activity in colorectal adenocarcinomas compared with *β-*, *γ-* and *α-*tocotrienol [21]. Moreover, δ-tocotrienol was found to be three times as effective as the chemotherapy agent tamoxifen in combating breast cancer, as reported by Nesaretnam *et al*. [22].

### 3.2. β-carotene content in crude palm oil after extraction by ionic liquids

**Figure 4.**
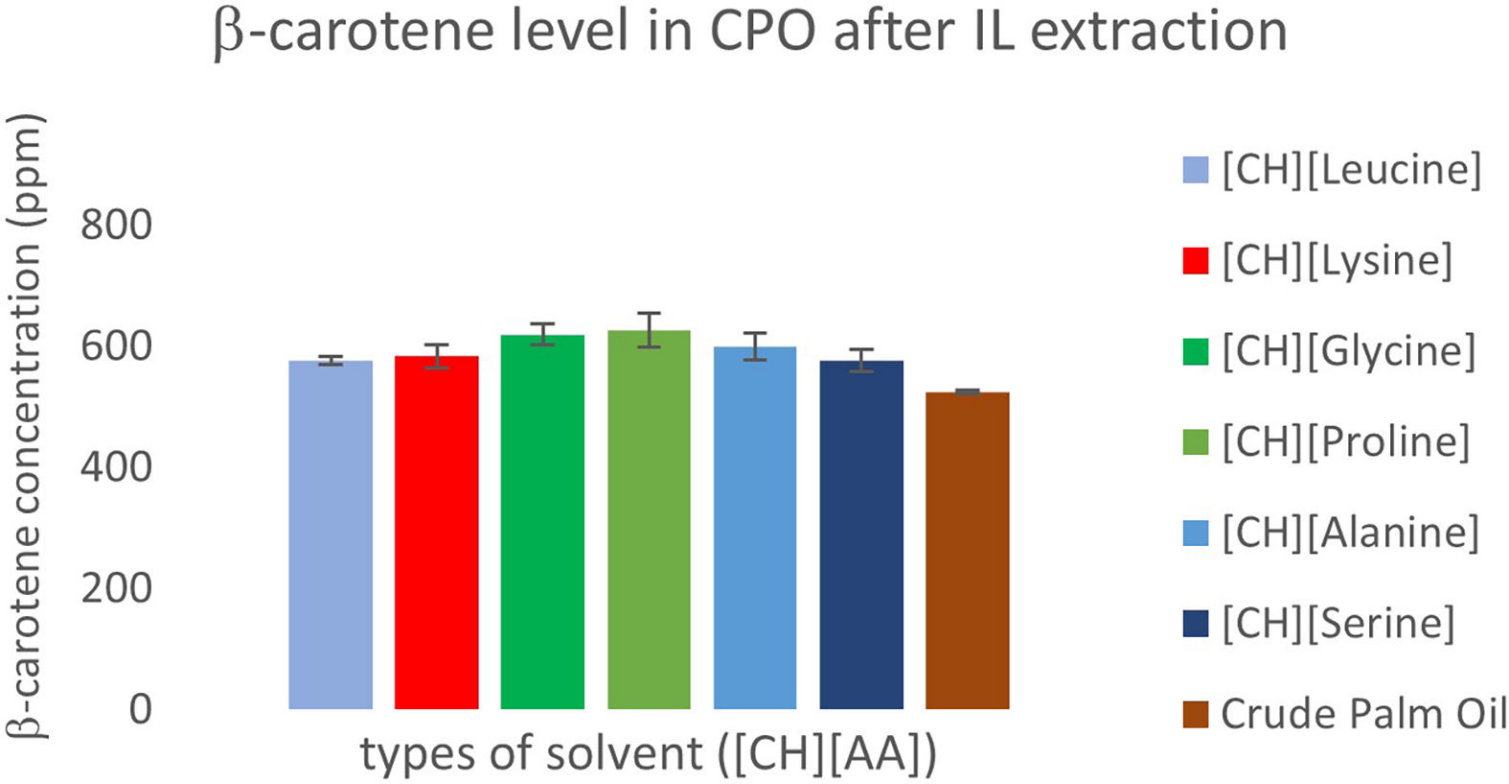
β-Carotene levels in CPO after extraction with ILs.

We evaluated the effectiveness of the ILs in extracting palm carotenes by analysing the residual carotene content in the CPO after extraction. Regardless of the anion type, the ILs were unable to extract carotenes from CPO. As shown in figure 4, the carotene content in pure CPO was 525 mg kg⁻¹. Post extraction, the carotene content in the oil slightly increased, with [CH][proline] showing the highest concentration at 627 mg kg⁻¹, followed by [CH][glycine] (620 mg kg⁻¹), [CH][alanine] (601 mg kg⁻¹), [CH][lysine] (583 mg kg⁻¹), [CH][serine] (577 mg kg⁻¹), and [CH][leucine] (576 mg kg⁻¹) . This increase may be attributed to the extraction of neutral oil and other components of CPO into the ILs, which concentrated the remaining carotenes in the oil post extraction.

Based on the observed trend in tocol extraction using [CH][AA] ILs, where less hydrophobic tocotrienol subtypes were extracted more efficiently, it is plausible that carotenes, being more hydrophobic than tocols owing to the absence of polar functional groups, exhibit limited affinity for the ILs. This probably accounts for their inability to be effectively extracted by [CH][AA] ILs.

### 3.3. Recyclability of ionic liquids

The recyclability of synthesized ILs is critical for industrial scaling and commercialization. Various methods exist for recycling ILs, including phase separation, extraction, distillation, adsorption, membrane separation and crystallization [23]. Given that [CH][AA] ILs are water-soluble while tocols are not, we used phase separation by mixing the IL with water after extraction, allowing the tocol layer to be siphoned off. The IL was then recovered by removing water through rotary evaporation.

Using phase separation with water, approximately 50% of the tocols were recovered from the IL (table 3). However, the recycled IL exhibited a diminished capacity for tocol extraction from CPO in subsequent uses, rendering it unsuitable for re-extraction.

**Table 3.** Extraction of individual tocols (mg kg^−1^) from crude palm oil (CPO) by recycled [CH][proline] IL. A: ([CH][proline] IL after extracting CPO in a 1 : 1 w/w ratio); B: (IL after phase separation to remove recover tocols from A); C: (IL B after extracting fresh CPO in a 1 : 1 w/w ratio).

tocol type	concentration (mg kg^−1^)		
	A	B	C
**δ-tocotrienol**	23.799 ± 1.134	13.582 ± 0.369	21.95 ± 3.541
**γ-tocotrienol**	92.092 ± 4.809	48.091 ± 1.628	72.99 ± 15.973
**α-tocotrienol**	22.335 ± 1.744	8.521 ± 0.460	15.138 ± 3.686
**α-tocopherol**	32.644 ± 3.646	10.041 ± 1.111	25.794 ± 9.325

### 3.4. Strengths, limitations and future perspectives

Our study’s primary strength lies in directly using neat IL and CPO for extraction. Almost all studies on liquid extraction of phytonutrients from palm oil using IL dilute CPO and IL into organic solvents like hexane and methanol to reduce viscosity and enable milder extraction conditions. Mixing CPO and IL with organic solvents may hinder industrial scalability as it still relies on flammable solvents, and accurately assessing IL extraction efficiency could be challenging. A review by Hoe *et al*. [5] provides an extensive comparison of the advantages and disadvantages of different phytonutrient extraction approaches from palm oil. For membrane processing, feedstock such as CPO is challenging owing to its viscosity and potential to cause membrane fouling. Our approach of heating and extracting with neat IL is well suited for handling CPO. While molecular distillation achieves high purity and recovery for volatile compounds, it requires significant energy input and specialized equipment, whereas our extraction approach operates under mild conditions, reducing energy consumption and preserving heat-sensitive phytonutrients. Similarly, adsorption achieves selective enrichment but depends on adsorbent regeneration and the use of solvents for desorption. By contrast, our approach eliminates the need for additional organic solvents, offering a distinct advantage.

While we demonstrated [CH][AA] ILs’ specificity in extracting tocols from CPO, a limitation of our work concerns the recyclability of ILs, warranting further optimization or exploration. Future research could explore various anion pairs to synthesize ILs, aiming to enhance tocol extraction yields, and investigate alternative methods for IL recycling to improve recyclability. Though [CH][AA] ILs are expensive, our method could reduce operational costs by obviating the need for flame-proof facilities and extensive clean-up steps associated with toxic solvents, potentially offering cost advantages over existing methods such as column chromatography and distillation for phytonutrient extraction. We recommend a comprehensive cost–benefit analysis to fully evaluate the economic viability of our extraction method.

## 4. Conclusion

Extraction of tocols and carotenes from CPO is challenging, and the use of novel extraction solvents such as ILs can aid in this regard. Using [CH][AA] ILs, we showed that the ILs are selective for extraction of tocols from CPO. We found that [CH][lysine] was the most effective IL, which removed > 200 mg kg^−1^ of tocol in a single step (a yield of approx. 25%). Between tocol subtypes, the ILs were particularly effective for extracting δ-tocotrienol potentially owing to its lower polarity compared with different tocol subtypes. Considering the non-volatility and biocompatibility of the ILs, it shows promise for industrial use in the enrichment of vitamin E derivatives from edible oils.

## Data Availability

Supplementary material is available online [24].
